# Transcriptomic Profiling of *Cutibacterium acnes* IA_1_—Infected Keratinocytes Reveal Hub Genes and CLR Pathway in Acne Pathogenesis

**DOI:** 10.3390/cimb48010034

**Published:** 2025-12-26

**Authors:** Jiawen Li, Fuxin Wang, Dangsheng Liu, Weichao Yang, Hao Sun, Mingfu Gao, Dawei Chen, Hui Xu

**Affiliations:** 1 Institute of Applied Ecology, Chinese Academy of Sciences, Shenyang 110016, China; lijiawen20@mails.ucas.ac.cn (J.L.); yangweichao@iae.ac.cn (W.Y.); haos@spaces.ac.cn (H.S.); gaomingfu@iae.ac.cn (M.G.); 2 University of Chinese Academy of Sciences, Beijing 100049, China; 3 Liaoning Institute for Drug Control, Shenyang 110036, China; wangfuxinfc@163.com; 4 School of Life Sciences and Biopharmaceutical Sciences, Shenyang Pharmaceutical University, Shenyang 110016, China; 13940081506@139.com; 5 School of Pharmacy, Shenyang Pharmaceutical University, Shenyang 110016, China

**Keywords:** acne vulgaris, *Cutibacterium acnes*, HaCaT keratinocytes, biomarkers, transcriptomics, inflammation, CLR pathway

## Abstract

Acne vulgaris is a prevalent chronic inflammatory skin disorder affecting over 85% of adolescents. Emerging evidence indicates that *Cutibacterium acnes* phylotype IA_1_ contributes to acne initiation and progression, yet its precise mechanisms in epidermal keratinocytes remain unclear. This study investigated *C. acnes* IA1’s effects on keratinocyte behavior using an in vitro HaCaT cell model. Cells were co-cultured with live *C. acnes* IA_1_ (CICC 10864) for 24 h. Transcriptomic profiling identified 769 differentially expressed genes (DEGs; adjusted *p* < 0.05, |log2FC| > 1), including 392 upregulated and 377 downregulated. The protein–protein interaction network analysis via Cytoscape revealed key hub genes (HNRNPA2B1, HNRNPM, RBM39). Enrichment analyses (GO, KEGG, Reactome, DO) highlighted significant involvement of the C-type lectin receptor (CLR) signaling pathway. Validation experiments showed cellular morphological changes, altered structure, and markedly elevated interleukin-6 (IL-6; *p* < 0.01), underscoring its role in inflammation. These findings suggest *C. acnes* IA_1_ drives acne pathogenesis by regulating hub genes that influence sebaceous gland inflammation, immune activity, and keratinocyte proliferation, positioning them as potential biomarkers for microbiome-targeted therapies. Limitations include the in vitro model’s lack of in vivo skin microenvironment complexity and use of only one representative IA_1_ strain.

## 1. Introduction

Acne vulgaris (referred to as acne hereafter), a multifactorial chronic inflammatory skin disorder with a global prevalence exceeding 85% among young adults, profoundly impacts the physical appearance and psychological well-being of adolescents [1,2]. Given its high prevalence, chronic recurrence, and potential to cause permanent scarring, acne represents a significant public health issue, frequently contributing to prolonged emotional distress, social isolation, and diminished self-esteem among affected individuals [3,4,5].

The pathogenesis of acne is multifaceted, involving intricate interactions among four primary factors: androgen-dependent hyper-seborrhea, follicular hyper-keratinization, inflammation, and microbial dysbiosis [1]. Central to acne pathogenesis is the role of *Cutibacterium acnes* (*C. acnes*, formerly *Propionibacterium acnes*), a gram-positive, anaerobic bacterium that colonizes the pilosebaceous follicle as part of the normal skin microbiota [6]. In healthy skin, *C. acnes* contributes to microbial homeostasis by producing antimicrobial peptides and maintaining an acidic pH that inhibits pathogenic invaders [7]. However, in acne-prone individuals, an imbalance in the skin microenvironment—such as increased sebum providing a nutrient-rich niche—can lead to the over-proliferation of specific *C. acnes* phylotypes, triggering inflammatory cascades [8]. Recent research has emphasized that acne development is not solely due to bacterial overgrowth but rather an imbalance among *C. acnes* phylotypes, with a loss of diversity serving as a key trigger for innate immune activation [9,10]. Among the six major phylotypes (IA_1_, IA_2_, IB, IC, II, III), phylotype IA_1_ demonstrates increased virulence and antibiotic tolerance, which is attributed to enhanced biofilm formation, stronger adhesion to keratinocytes, and elevated secretion of pro-inflammatory factors such as extracellular vesicles, lipases, and porphyrins [11,12,13]. Studies have shown a predominance of phylotype IA_1_ in acne lesions compared to healthy skin, where it alters the natural stratum corneum lipid ratio, invades follicular keratinocytes, dysregulates epidermal barrier function, and induces cytokine release and immune cell recruitment [10,12,14,15,16]. Moreover, recent studies have shown that the phylotype IA_1_’s interaction with follicular keratinocytes and cells in the sebaceous duct amplifies inflammation through pattern recognition receptors, such as Toll-like receptors (TLRs) and C-type lectin receptors (CLRs), leading to NF-κB pathway activation and production of interleukins (e.g., IL-6, IL-8) and tumor necrosis factor-alpha (TNF-α) [15,17,18,19]. However, there remains a gap in understanding the biological effects of *C. acnes* IA_1_ on epidermal keratinocytes, particularly with regard to the precise host transcriptional responses induced by this phylotype.

This investigation aims to delineate the regulatory mechanisms of *C. acnes* IA_1_ in acne pathogenesis, with a focus on identifying core genes, biological functions, and signaling pathways through integrated transcriptomics and network pharmacology. By constructing a PPI network and performing enrichment analyses, we seek to pinpoint hub genes that modulate sebaceous gland inflammation, immune cell activity, and keratinocyte proliferation. We selected *C. acnes* CICC 10864 (equivalent to ATCC 6919) as a representative IA_1_ reference strain, widely utilized in acne research for its well-characterized virulence profile.

## 2. Materials and Methods

### 2.1. Cell Culture

The human keratinocyte cell line HaCaT was purchased from Shanghai Chuanqiu biotechnology company. HaCaT cells were maintained in Dulbecco’s modified Eagle’s medium (DMEM) without antibiotics and supplemented with 10% fetal bovine serum. Cells were cultured at 37 °C in a humidified incubator under a 5% CO_2_ atmosphere.

### 2.2. Bacterial Culture

*C. acnes* (CICC 10864) belonging to phylotype IA_1_ was purchased from China Center of Industrial Culture Collection (Beijing, China) and cultured on Reinforced Clostridial Agar Medium (RCM) at 37 °C under anaerobic conditions using anaerobic atmosphere generation systems (Mitsubishi Gas Chemical Company, Inc., Tokyo, Japan). Briefly, plates or slants were placed in rectangular jars containing an AnaeroPack and incubated at 37 °C. Then, *C. acnes* IA_1_ (CICC 10864) was inoculated onto an agar slant medium and cultured for 24 h, after which the growth was harvested using an inoculating loop and washed with a 0.85% saline solution. The resulting bacterial pellet was resuspended in 0.85% saline to obtain a uniform bacterial suspension and adjusted by dilution to an absorbance of OD_625nm_ = 1.0, corresponding to approximately 1 × 10^9^ CFU/mL. The bacterial cell concentration was accurately determined using a pre-established standard curve correlating OD_625nm_ readings with cell counts (y = 0.9541x + 0.0358, R^2^ = 0.968).

### 2.3. Co-Culture of Keratinocytes with C. acnes IA_1_

At first, HaCaT cells were seeded in 96-well plates and 60 mm culture dishes at a seeding density of 1 × 10^5^ cells per well and 5 × 10^6^ cells per dish, respectively. The cells were allowed to attach for 24 h, which would grow to reach 95% confluency. Then, the HaCaT cells were treated with *C. acnes* IA_1_ (CICC 10864) at multiplicity of infection (MOI) for 24 h. Briefly, the culture medium was replaced with a different concentration of bacterial suspension obtained as above. Then, HaCaT and *C. acnes* IA_1_ (CICC 10864) were co-cultured at 37 °C/5% CO_2_ for 24 h.

### 2.4. RNA Extraction

Total RNA was extracted from the cell using TRIzol Reagent (Beyotime Biotechnology, Shanghai, China) and purified using an RNA Purification Kit (Shanghai Majorbio, Shanghai, China) according the manufacturer’s instructions. The concentration and purity of the isolated RNA were assessed using a Nanodrop 2000 spectrophotometer (NanoDrop Technologies, Shanghai, China). RNA integrity was evaluated through agarose gel electrophoresis, and the RQN value was determined using a 5300 Fragment Analyzer System (Agilent Technologies, Beijing, China). For individual sequencing library construction, a minimum of 1 μg total RNA is required, with a concentration of at least 30 ng/μL, an RQN value greater than 6.5, an OD260/280 ratio ranging between 1.8 and 2.2, OD260/OD230 greater than 2.0, and 28/23S rRNA band brightness exceeding that of 18/16S rRNA.

### 2.5. Library Preparation and Sequencing

RNA purification, reverse transcription, library construction, and sequencing were performed at Shanghai Majorbio Bio-pharm Biotechnology Co., Ltd. (Shanghai, China) according to the manufacturer’s instructions. The XX RNA-seg transcriptome library was prepared following Illumina Stranded mRNA Prep, Ligation (Illumina, Inc., Shanghai, China) using 1 μg of total RNA. Shortly, messenger RNA was isolated according to the polyA selection method by oligo (dT) beads and then fragmented by a fragmentation buffer. Next, double-stranded cDNA was synthesized using an Invitrogen^TM^ SuperScript double-stranded cDNA synthesis kit (Thermo Fisher Scientific, Shanghai, China) with random hexamer primers. Then, the synthesized cDNA was subjected to end-repair, phosphorylation, and adapter addition according to the library construction protocol. Libraries were size selected for cDNA target fragments of 300 bp on 2% Low Range Ultra Agarose followed by PCR amplified using Phusion DNA polymerase (NEB) for 15 PCR cycles. After quantification by Qubit 4.0, the sequencing library was performed on the NovaSeq X Plus platform (PE1 50) using the Illumina NovaSeg Reagent Kit (Illumina, Inc.).

### 2.6. Differential Expression Gene Analysis

To identify differentially expressed genes (DEGs) between two distinct samples, the expression level of each transcript was estimated using the transcripts per million (TPM) method. Gene abundance quantification was carried out with RSEM. Subsequently, differential expression analysis was conducted using DESeq2. Genes exhibiting a |log2 fold change (FC)| ≥ 1 and a false discovery rate (FDR) < 0.05 (as determined by DESeq2) were considered significantly differentially expressed.

### 2.7. Functional Enrichment Analyses of Hub Genes

Functional enrichment analysis including GO and KEGG were performed to identify which DEGs were significantly enriched in GO terms and which metabolic pathways had a Bonferroni-corrected *p*-value ≤ 0.05 compared with the whole-transcriptome background. GO functional enrichment and KEGG pathway analysis were carried out by Goatools (version 0.12.1) and Python scipy (version 1.10.1) software, respectively.

### 2.8. PPI Network Construction and Key Genes Identification

To investigate the interactions among DEGs and identify potential key genes, a protein–protein interaction (PPI) network was constructed using data from the STRING database. Only interactions with a combined score ≥ 0.7 (indicating high confidence) were retained for network construction. This constructed PPI network was later visualized using Cytoscape Software (version 3.10.2). We then calculated the top 20 significant nodes within the PPI network based on their degrees using cytohubba, an extension of Cytoscape. These 20 calculated nodes were recognized as candidate key genes (hub genes) associated with *C. acnes*.

### 2.9. Host Cell Viability Assay

HaCaT cells seeded in 96-well plates were co-cultured with *C. acnes* IA_1_ (CICC 10864) at MOIs of 200:1, 150:1, 100:1, 50:1, 10:1, and 1:1. Meanwhile, the corresponding series of bacterial suspension concentrations cultured without keratinocytes served as blank controls, whereas keratinocytes cultured without bacteria were defined as negative controls. After 24 h, cell viability was determined by using cell-counting kit 8 (CCK-8, Beyotime Biotechnology). Firstly, the culture medium was removed by aspiration and centrifugated at 13,000× *g* for 5 min. The supernatant was then collected and stored at −80 °C for further experimental use. Next, a CCK-8-containing medium was added to each well and incubated for 1 h in a CO_2_ incubator at 37 °C. The absorbance value was measured on a Spark Multimode Microplate Reader (Tecan Trading AG) with a test wavelength of 450 nm and a reference wavelength of 630 nm.

(1) $$Cell\;viability\left(\%\right)=\frac{\left(A_{450}^{s}-A_{630}^{s}\right)-\left(A_{450}^{bc}-A_{630}^{bc}\right)}{\left(A_{450}^{nc}-A_{630}^{nc}\right)}$$

A^S^: The absorbance of keratinocytes co-cultured with bacteria cells.
A^nc^: The absorbance of keratinocytes cultured without bacteria cells.
A^bc^: The absorbance of bacteria cells cultured without keratinocytes.

### 2.10. Pro-Inflammatory Cytokine Using ELISA Assay

The supernatant collected above (Section 2.9) was used to determine the IL-6 secretion without dilution using an ELISA immunoassays kit (Beijing Shengyan Biology, Beijing, China). The secretion of the pro-inflammatory cytokine IL-6 was determined by ELISA according to the manufacturer’s protocol, with a linearity range of 1.5–48 pg/mL. Three technical replicates were performed for each sample. The absorbance value of each sample was measured at 450 nm using a Spark Multimode Microplate Reader (Tecan Trading AG, Shanghai, China).

### 2.11. Calculation of the Cytokine Secretion

In order to reduce any variation due to the differences in cell density, the normalized cytokine secretion values were adjusted by the cell viability using the following equation:

(2) $$Normalized\;cytokine\;secretion=\frac{cytokine\;secretion(pg/mL)}{cell\;relative\;viability}$$

The cells not treated with *C. acnes* IA_1_ were designated as the control group, which had a viability of 1, and the relative viability of the treated groups was compared to that of the control group.

### 2.12. Statistical Analysis

All the statistical analyses were performed using GraphPad Prism Version 9.0.0 (GraphPad Software Inc., San Diego, CA, USA). Data were expressed as means ± standard deviation (SD) of three biological replicates or three independent experiments. Paired *t*-test or ordinary one-way analysis of variance (ANOVA) with Dunnett’s multiple comparisons test was used to identify significant variations. *p* < 0.05 was considered to indicate a statistically significant difference.

### 2.13. Bacterial Cell Tracing

To analyze the presence of bacteria, *C. acnes* IA_1_ (CICC 10864) was stained with CytoTrace™ UltraGreen (AAT Bioquest, Pleasanton, CA, USA) in accordance with the manufacturer’s instructions. In brief, the bacterial cells were treated with CytoTrace™ UltraGreen for 30 min at 37 °C and then added to HaCaT cells at an MOI of 100:1 for 24 h. Then, the floating bacteria were removed by washing 3 times with PBS. The cells were fixed on ice with a 4% formaldehyde solution in PBS for 15 min and then permeabilized with 0.5% Triton X-100 in PBS for 10 min at room temperature. Subsequently, the cells were stained by Phalloidin-AF594 (Cohesion Biosciences, London, UK) for 20 min, followed by Hoechst 33342 (Beyotime Biotechnology) for 5 min according to the manufacturer’s instructions. The samples were observed by using ZEISS Axiovert 5 fluorescence microscopy (ZEISS, Jena, Germany).

### 2.14. Bacterial Quantification

To quantify the viability of bacteria attached to the host cell, bacterial counting by qPCR was performed as follows. HaCaT cells were cultured on 60 mm dishes, followed by treatment with *C. acnes* at an MOI of 100:1 for 24 h. The culture medium was collected. Subsequently, the cells were gently washed three times with 1 mL of PBS, then the washing buffer was collected and combined with the culture medium to obtain the non- associated bacteria. In addition, the keratinocyte cells were lysed by adding 2 mL of sterile distilled water, incubating for 5 min at room temperature to induce cell swelling, and pipetting up and down gently until complete cell rupture was achieved to release the potentially adherent or invasive bacteria, and the extract was collected. Total DNA was isolated from the bacteria cells by using the GeneJET™ Genomic DNA Purification Kit (Thermo Fisher Scientific) according to the manufacturer’s instructions. DNA concentration and purity were determined by standard spectrophotometric methods using a NanoVue™ Plus Spectrophotometer (Richmond Scientific, Lancashire, UK) with an OD260/280 ratio ranging between 1.8 and 2.0. Real-time PCR targeted on a bacterial 16S rRNA gene was carried out to detect the copy numbers of the samples with primers specific for *C. acnes* (PA1-129F: 5′- GACTTTGGGATAACTTCAGGAAACTG -3′, PA1-238R: 5′- CTGATAAGCCGCGAGTCCAT -3′) using the SYBR Green PCR master mix (Sparkjade, Jinan, China) and the Bio-Rad CFX96 Real-Time PCR System (Bio-Rad, Hercules, CA, USA). The thermal cycling conditions consisted of an initial denaturation at 94 °C for 3 min, followed by 40 cycles of 94 °C for 15 s, 60 °C for 20 s, and 72 °C for 30 s; a final hold at 95 °C for 10 s; and melt curve analysis. The amplification specificity was confirmed by melting curve analysis. The data were calculated by using the standard curve method for absolute quantification (y = −3.738x + 40.337, E = 85.4%, R^2^ = 0.996, slope = −3.730). In addition, the preparation of the standard template was as follows. DNA of the reference strain *C. acnes* CICC10864 was used as the template for full-length amplification of the 16S rDNA gene fragment using bacterial universal primers (27F: 5′-AGAGTTTGATCMTGGCTCAG-3′; 1429R: 5′-TACCTTGTTACGACTT-3′). The amplification was carried out under the following conditions: initial denaturation at 95 °C for 2 min; 30 cycles of 95 °C for 30 s, 50.4 °C for 30 s, and 72 °C for 90 s; a final extension at 72 °C for 10 min; and holding at 4 °C. PCR amplicons were run on 1% agarose TAE gels. Then, 16S rDNA at 1500 bp were extracted by following the GeneJET™ Gel Extraction Kit manufacturer’s protocol (Thermo Fisher Scientific), and the purity and concentration of extracted DNA were measured by using the NanoVue™ Plus Spectrophotometer (Richmond Scientific) with an OD260/280 ratio ranging between 1.8 and 2.0. Then, the copy number was calculated and corrected. The extracted 16S rDNA were used as a standard template for construction of standard curves.

## 3. Results

### 3.1. Viability of HaCaT Cells After Co-Culturing with Different Amounts of C. acnes IA_1_

**Figure 1:**
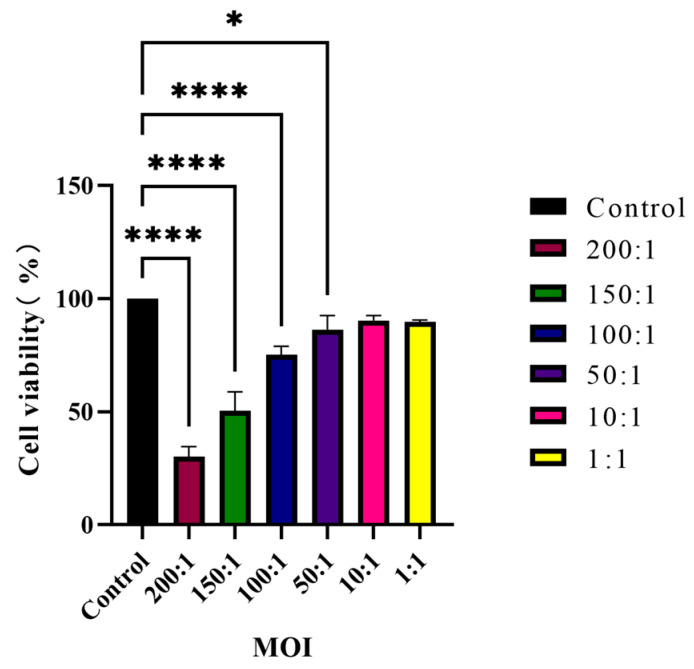
Viability of HaCaT cells co-cultured with *C. acnes* IA_1_ (*n* = 3 independent experiments, * *p* < 0.05 and **** *p* < 0.0001).

The cytotoxicity of varying concentrations of *C. acnes* IA_1_ (CICC 10864) on keratinocytes was assessed using the Cell Counting Kit-8 (CCK-8) method. HaCaT cells were exposed to different ratios of *C. acnes* IA_1_ (CICC 10864), and it was observed that cell viability was approximately 75% (mean ± SD: 75.23 ± 3.65%) at an MOI of 100:1 compared to uninfected controls (set at 100%), indicating moderate cytotoxicity suitable for modeling acne-related inflammation without widespread cell death. Higher MOIs (150:1 and 200:1) reduced viability to 50.45 ± 8.38% and 30.03 ± 4.58%, respectively, suggesting significant cytotoxic effects, while lower MOIs (1:1 to 50:1) showed minimal impact (>90% viability). The MOI of 100:1 was selected for subsequent experiments to balance infection-induced cellular responses with sufficient cell survival, consistent with prior studies of *C. acnes*–keratinocyte interactions. Results are summarized in Figure 1, which illustrates the dose-dependent decline in viability, with statistical significance confirmed via one-way ANOVA (*p* < 0.001).

### 3.2. PCA Analysis and DEG Identification

**Figure 2:**
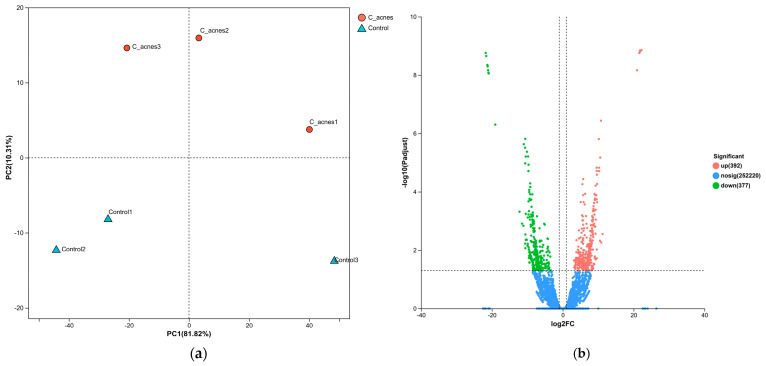
DEG analysis of our RNA–Seq data. (**a**) PCA analysis. (**b**) Volcano plot showing the results of DEG analysis (DESeq2 (adjusted *p* < 0.05, |log2FC| > 1)).

In this study, principal component analysis (PCA) was conducted on the gene expression values across all samples. As depicted in Figure 2a, the PCA plot delineates two distinct clusters corresponding to the control and *C. acnes* IA_1_ groups. The control samples exhibit a tendency to form compact clusters, whereas the *C. acnes* group demonstrates less pronounced clustering, potentially attributable to variations in the conditions of HaCaT cells.

A total of 769 DEGs were identified, meeting the criteria of an adjusted *p*-value < 0.05 and absolute log2FC > 1. Among these DEGs, 392 were upregulated, and 377 were downregulated. Figure 2b presents a volcano plot depicting the differentially expressed genes (DEGs), where upregulated genes are indicated by red dots and downregulated genes by blue dots. Genes exhibiting no significant change in expression are represented by gray dots. The top 10 upregulated and downregulated DEGs are detailed in Table 1. Quality control metrics confirmed comparable sequencing quality between groups: total reads per sample ranged from 41.9 to 48.1 million, with mapping rates to the human genome (GRCh38) ranging from 97.78% to 98.31% (Table S1).

### 3.3. Functional Enrichment Analysis

**Figure 3:**
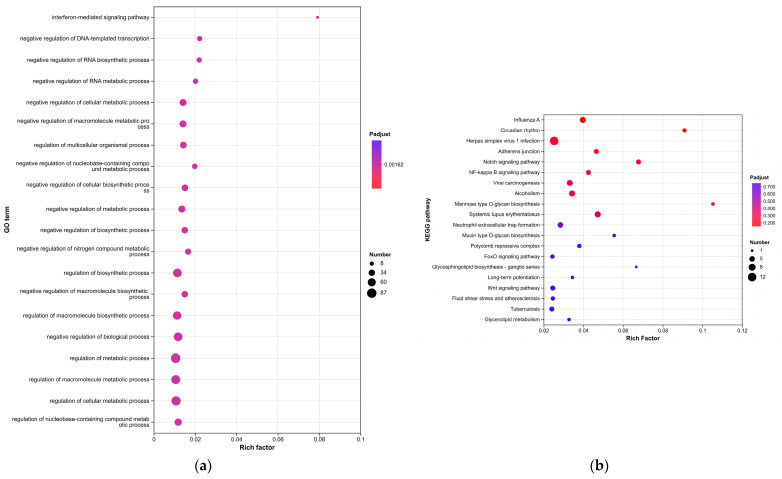

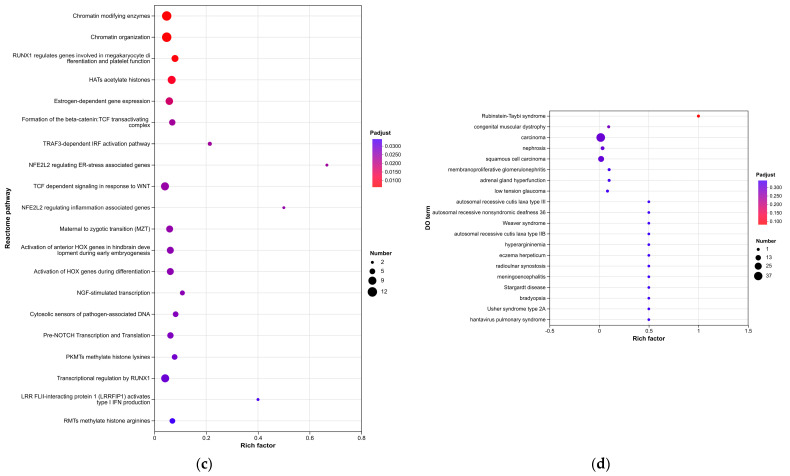
(**a**) GO analysis; (**b**) KEGG analysis; (**c**) Reactome analysis; (**d**) DO analysis.

To elucidate the biological classification of the identified differentially expressed genes (DEGs), we performed enrichment analyses utilizing Gene Ontology (GO) and the Kyoto Encyclopedia of Genes and Genomes (KEGG) pathways. Figure 3 displays the enrichment analysis results for biological processes, which revealed that the DEGs were specifically enriched in the interferon-mediated signaling pathway and showed negative regulation of DNA-templated transcription, negative regulation of the RNA biosynthetic process, negative regulation of the RNA metabolic process, negative regulation of the cellular metabolic process, negative regulation of the macromolecule metabolic process, regulation of the multicellular organismal process, negative regulation of the nucleobase-containing compound metabolic process, negative regulation of the cellular biosynthetic process, and negative regulation of the metabolic process (Figure 3a). The top 10 GO terms for biological processes, cellular components, and molecular functions are listed in Table 2.

The results of the KEGG pathway enrichment analysis of core target genes in HaCaT cells treated with *C. acnes* IA_1_ revealed that influenza A, circadian rhythm, herpes simplex virus 1 infection, adherents junction, notch signaling pathway, NF-kappa B signaling pathway, viral carcinogenesis, alcoholism, mannose type O-glycan biosynthesis, systemic lupus erythematosus, neutrophil extracellular trap formation, mucin type O-glycan biosynthesis, polycomb repressive complex, FoxO signaling pathway, glycosphingolipid biosynthesis, long-term potentiation, Wnt signaling pathway, fluid shear stress and atherosclerosis, tuberculosis, and glycolipid metabolism contained high rates of enriched genes and large *p*-values, as shown in Figure 3b.

The Reactome enrichment analysis of core target genes in HaCaT cells treated with *C. acnes* IA_1_ demonstrated significant enrichment across multiple pathways, including chromatin-modifying enzymes, chromatin organization, RUNX1 (which regulates genes involved in megakaryocyte differentiation and platelet function), HATs acetylate histones, estrogen-dependent gene expression, formation of the beta-catenin: TCF transactivating complex, TRAF3-dependent IRF activation pathway, NFE2L2-regulating ER-stress associated genes, TCF-dependent signaling in response to WNT, and NFE2L2-regulating inflammation-associated genes. These pathways exhibited high gene enrichment rates coupled with significant *p*-values, as depicted in Figure 3c.

The Disease Ontology (DO) enrichment analysis performed on core target genes from HaCaT cells treated with *C. acnes* IA_1_ revealed that several pathways demonstrated high enrichment rates and statistically significant *p*-values. These pathways included Rubinstein–Taybi syndrome, congenital muscular dystrophy, carcinoma, nephrosis, squamous cell carcinoma, membranoproliferative glomerulonephritis, adrenal gland hyperfunction, low-tension glaucoma, autosomal recessive cutis laxa type III, and autosomal recessive nonsyndromic deafness 36. The detailed results are illustrated in Figure 3d.

**Figure 4:**
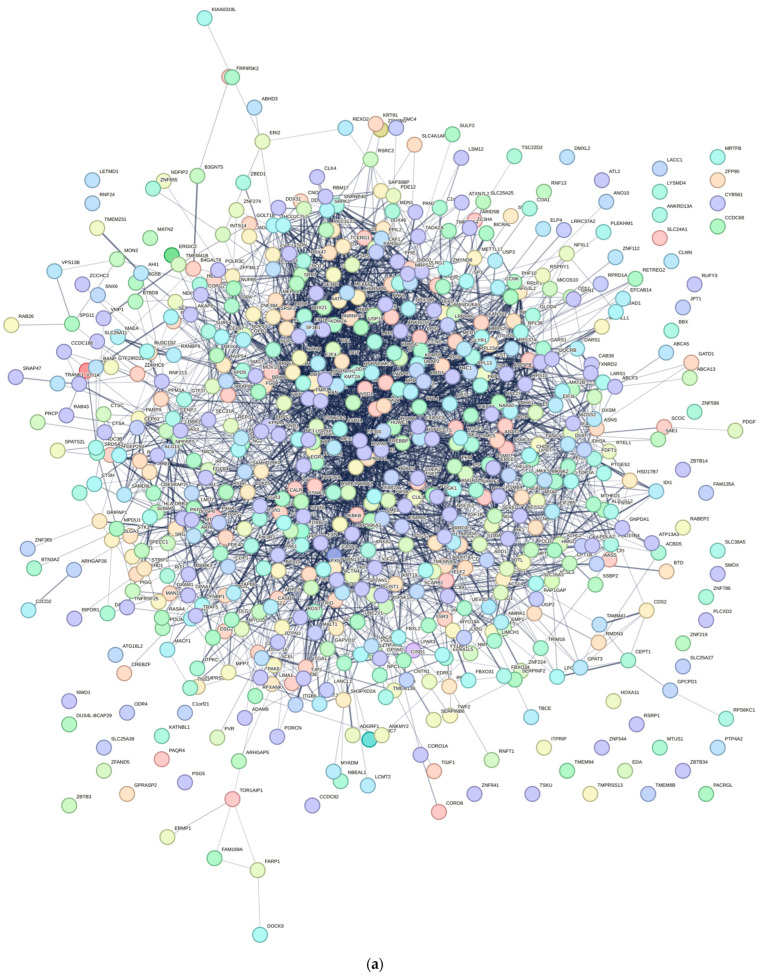

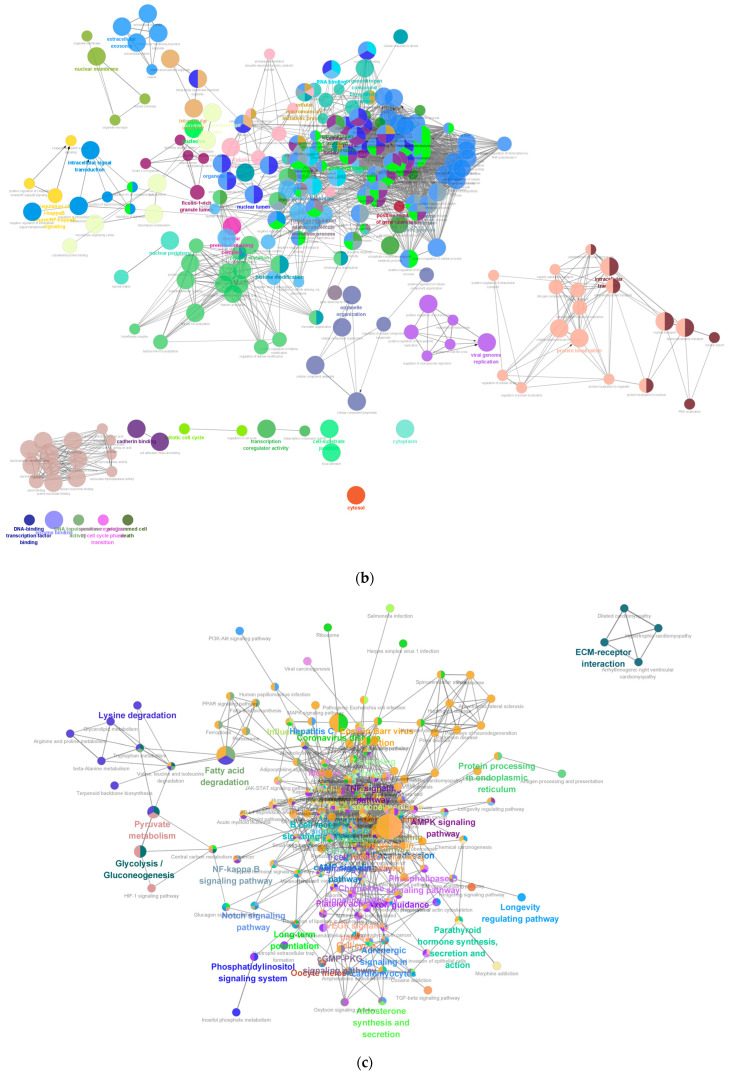
(**a**) PPI network of differentially expressed mRNAs; (**b**) clueGO analysis of genes in PPIs that had differentially expressed mRNAs associated with acne; (**c**) clueGO–KEGG analysis of genes in PPIs that had differentially expressed mRNAs associated with acne.

Additionally, an interaction network among the KEGG pathways was constructed utilizing the clueGO software (version 2.5.10), as illustrated in Figure 4c. Numerous differentially expressed genes (DEGs) were identified as participants in multiple pathways.

To address the extensive list of enriched pathways, we grouped them into functional categories for better prioritization and interpretation (Table 3). The top enriched pathways from GO, KEGG, Reactome, and DO analyses were categorized as follows:

- Immune Response: Pathways such as p53 signaling (*p* < 0.01) highlighted inflammatory cytokine regulation, aligning with IL-6 upregulation observed experimentally.
- Chromatin Remodeling: DNA repair pathways like homologous recombination and nucleotide excision repair (q-value < 0.05) suggest epigenetic modifications in response to bacterial stress.
- Metabolic Processes: Biosynthetic routes including ubiquinone biosynthesis and aminoacyl-tRNA biosynthesis indicate metabolic shifts supporting cellular adaptation.
- RNA Processing and Transcription: Dominant categories with spliceosome, RNA transport, and mRNA surveillance (enriched in 392 upregulated DEGs), underscoring the role of hub genes in RNA splicing.
- Protein Degradation and Cell Cycle: Proteasome and ubiquitin-mediated proteolysis pathways link to keratinocyte proliferation control.

These groupings emphasize the CLR signaling pathway’s integration with RNA and immune modules, providing a focused framework for acne pathogenesis.

### 3.4. PPI Network Analysis

**Figure 5:**
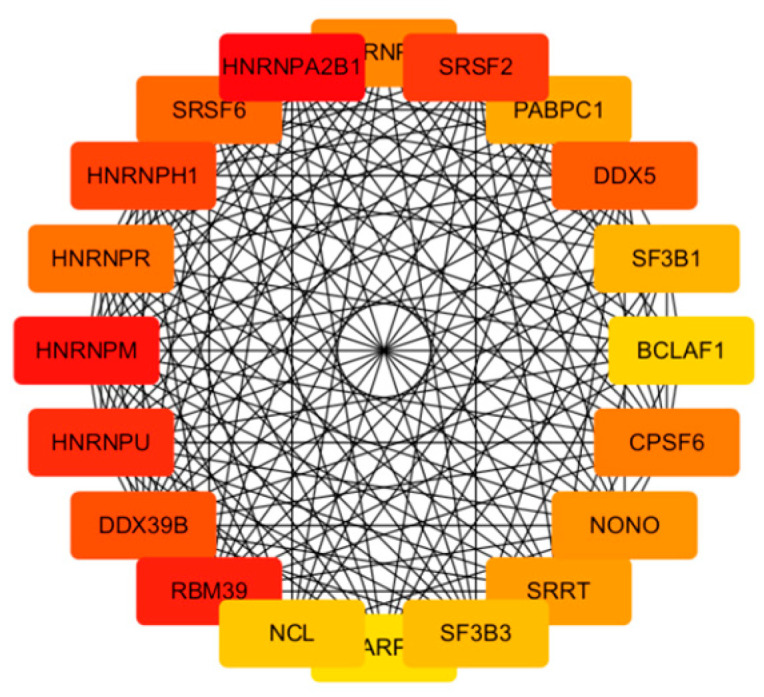
Network diagram in top 20 hub genes.

The protein–protein interaction (PPI) network comprised 585 nodes and 3405 edges, as illustrated in Figure 4a. The network exhibited an average neighbor count of 11.641 and a local clustering coefficient of 0.136. Notably, the proteins HNRNPA2B1, HNRNPM, and RBM39 demonstrated the highest combined scores, with nodal degrees of 51, 41, and 55, respectively. Top 20 hub genes were also identified; results are shown in Figure 5 and Table 4. Furthermore, clueGO and clueGO-KEGG analysis were performed, and the genes within the protein–protein interaction that are associated with differentially expressed mRNAs in acne are illustrated in Figure 4b,c. The results highlight potential pathways and functions associated with acne development.

### 3.5. GO and KEGG Enrichment Analyses of Hub Genes

**Figure 6:**
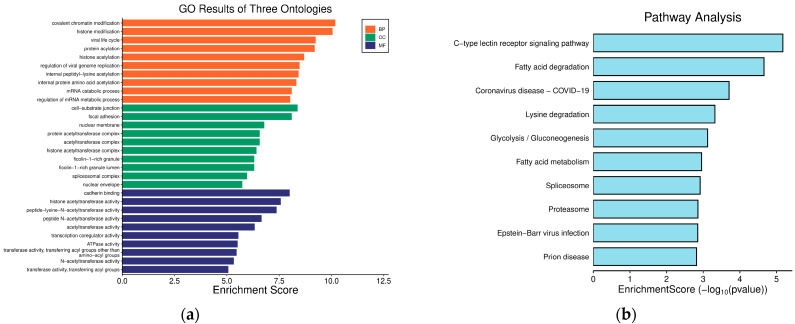
Bar graph of hub genes GO and KEGG enrichment analyses. (**a**) GO analysis; (**b**) KEGG analysis.

In GO analysis, the hub genes were predominantly enriched in covalent chromatin modification, histone modification, protein acylation, the cell–substrate junction, focal adhesion, the protein acetyltransferase complex, cadherin binding, histone acetyltransferase activity, and peptide-lysine-N-acetyltransferase activity. In KEGG pathway analysis, hub genes were mainly enriched in the C-type lectin receptor signaling pathway, fatty acid degradation, and coronavirus disease—COVID-19. Figure 6 presents the summary of GO and KEGG enrichment analyses.

### 3.6. Morphological and Cell Structure Changes in HaCaT Cells with C. acnes IA_1_

**Figure 7:**
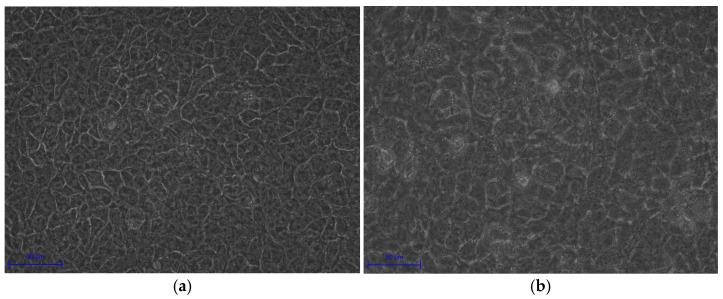
Morphological changes in cells infected by *C. acnes* IA_1_. (**a**) Control of HaCaT cells; (**b**) HaCaT cells co-cultured with *C. acnes* IA_1_.

HaCaT cells co-cultured with *C. acnes* IA_1_ (CICC 10864) at an MOI of 100:1 displayed significant morphological alterations, characterized by cell swelling and indistinct boundaries (Figure 7), indicating notable changes in both cell volume and boundary definition compared to the negative control. Cell swelling was quantified by measuring cell area and circumference with ZEISS Labscope software (version 4.5) and comparisons determined by a paired *t*-test (Table 5). The presence of *C. acnes* IA_1_ (CICC 10864) induced contraction in the epidermal cells, leading to membrane folding and consequently producing a more irregular cellular shape. This finding is consistent with the viability study indicating that *C. acnes* IA_1_ (CICC 10864) does not highly significantly increase cell mortality but does affect the keratinocytes to a certain extent.

### 3.7. The Attachment of C. acnes IA_1_ to HaCaT Cells

**Figure 8:**
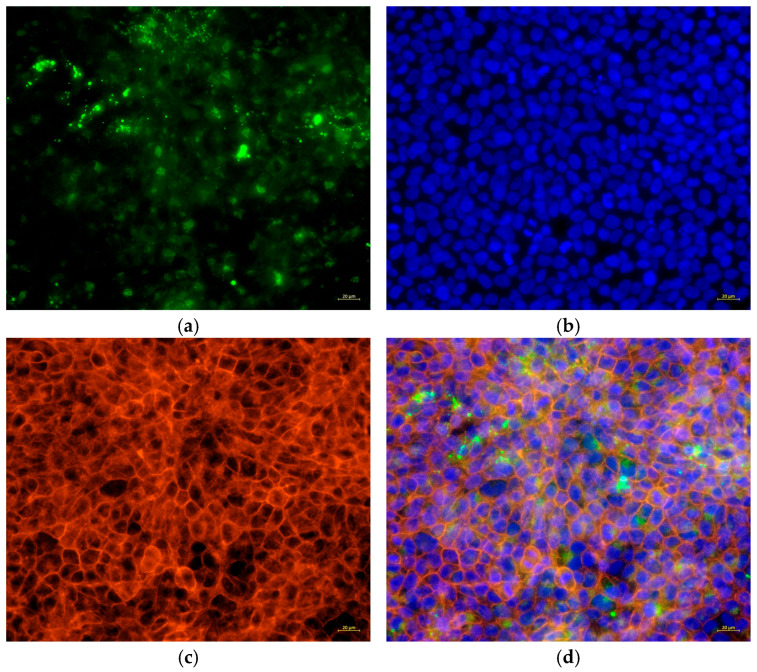
Cell structures fluorescently stained and co-cultured with *C. acnes* IA_1_. (**a**) *C. acnes* attached to host cells with fluorescent staining; (**b**) nuclear fluorescent staining; (**c**) cytoskeleton fluorescent staining; (**d**) merge.

In the experiment, to investigate the attachment between HaCaT cells and bacterial cells, HaCaT cells were co-cultured with green fluorescently labeled *C. acnes* IA_1_ (CICC 10864) at a multiplicity of infection (MOI) of 100:1 for 24 h, and then the cells were washed with PBS to remove the non-associated bacterial cells. In the subsequent steps, the nuclei and cytoskeletons of the keratinocytes were labeled with blue and red fluorescence, respectively, to delineate the cellular outline of the keratinocytes. Figure 8 illustrates that *C. acnes* IA_1_ (CICC 10864) was still observed on the surface of HaCaT cells after three washes with PBS. This suggests that *C. acnes* can adhere to, and potentially invade, host cells. To further test this hypothesis, HaCaT cells were lysed, and the associated bacterial cells were quantified using qPCR. The results revealed that some bacterial cells adhered to or even invaded keratinocytes (Table 6).

### 3.8. Pro-Inflammatory Cytokine Test

**Figure 9:**
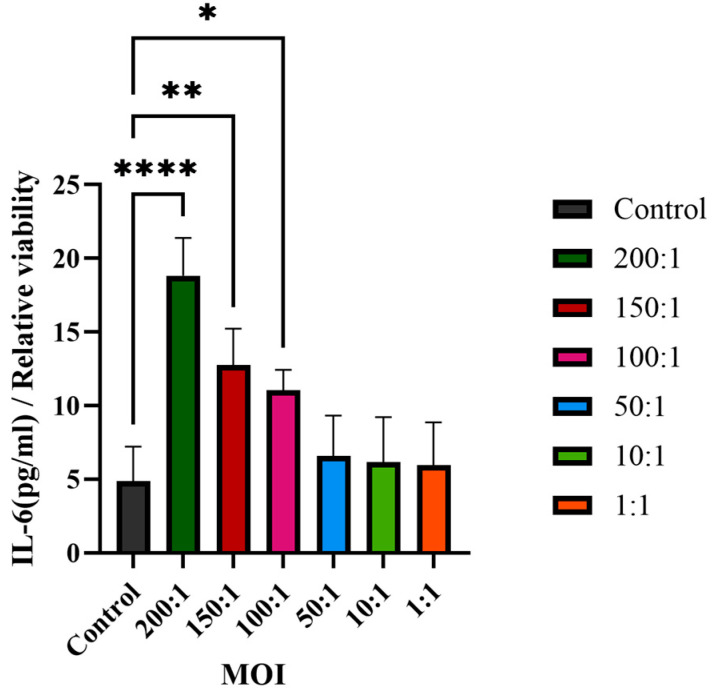
IL-6 secretion in HaCaT cells co-cultured with *C. acnes* IA_1_ (*n* = 3 independent experiments, * *p* < 0.05, ** *p* < 0.01, and **** *p* < 0.0001).

Based on transcriptome data showing significant upregulation of IL6 signaling pathways (Figure 3b, Table 1), we quantified IL-6 secretion. This highlights that the normalization of cytokine secretion to cell viability values can reveal relevant biological responses. As the concentration of *C. acnes* increased from an MOI of 100:1 to 200:1, there was a corresponding concentration-dependent increase in the secretion of IL-6. Figure 9 presents an overview of the secretion of IL-6 in HaCaT cells co-cultured with varying ratios of *C. acnes* IA_1_ (CICC 10864), with statistical significance confirmed via one-way ANOVA.

## 4. Discussion

This study provides novel insights into the molecular mechanisms underlying *Cutibacterium acnes* IA_1_–driven acne pathogenesis through transcriptomic profiling of infected HaCaT keratinocytes. By integrating RNA sequencing with network pharmacology, we identified 769 differentially expressed genes (DEGs), with 392 upregulated and 377 downregulated, reflecting profound transcriptional reprogramming in response to *C. acnes* IA_1_. The distinct clustering observed in the principal component analysis (PCA) underscores the robust impact of bacterial infection on keratinocyte gene expression, likely driven by inflammatory signaling and cellular stress responses [20]. These findings align with prior reports that *C. acnes* IA_1_, a virulent phylotype, triggers innate immune activation in acne-prone skin, contributing to chronic inflammation and lesion formation [15,21].

Central to our findings are the hub genes HNRNPA2B1, HNRNPM, and RBM39, identified through the protein–protein interaction (PPI) network analysis as key regulators of acne pathogenesis. HNRNPA2B1, a heterogeneous nuclear ribonucleoprotein, modulates RNA splicing and stability, and its upregulation in infected keratinocytes may enhance the expression of pro-inflammatory transcripts, such as those encoding cytokines like IL-6 [22]. This is consistent with our observation of significantly elevated IL-6 levels (*p* < 0.01), a cytokine known to promote keratinocyte proliferation and sebocyte differentiation in acne [23]. HNRNPM, involved in alternative splicing, likely contributes to follicular hyper-keratinization by dysregulating genes associated with keratinocyte differentiation, a hallmark of comedogenesis. Similarly, RBM39, a splicing factor interacting with U2AF65, may amplify inflammatory signaling by modulating C-JUN phosphorylation, mimicking pathogen-associated molecular patterns (PAMPs) that activate innate immunity [24]. These hub genes collectively regulate sebaceous gland inflammation, immune cell recruitment, and epidermal proliferation, positioning them as promising biomarkers for acne severity and potential therapeutic targets. For instance, elevated HNRNPA2B1 expression could serve as a diagnostic indicator in acne lesions, enabling personalized interventions targeting RNA processing pathways [20].

Enrichment analyses, grouped by functional categories (Table 3), revealed a predominance of RNA processing and immune response pathways consistent with *C. acnes*-induced inflammation. These analyses further identified the C-type lectin receptor (CLR) signaling pathway as a key mediator of *C. acnes* IA_1_–induced inflammation. CLRs, expressed on keratinocytes and immune cells, recognize bacterial glycans, activating NF-κB and MAPK pathways to drive cytokine production [25]. Our KEGG results, which also enriched NF-κB signaling, suggest a synergistic mechanism where *C. acnes* IA_1_ exploits CLR-mediated recognition to amplify inflammatory cascades, contributing to the chronicity of acne lesions [26]. Additionally, enrichment of interferon-mediated signaling and Notch signaling indicates broader immune modulation, with interferons enhancing antiviral-like responses against bacterial PAMPs and Notch regulating epidermal differentiation [27]. Reactome and Disease Ontology (DO) analyses further linked DEGs to chromatin modification and dermatological conditions, reinforcing acne’s inflammatory nature akin to psoriasis or eczema [28].

Experimental validation corroborated these transcriptomic insights. Morphological changes in infected HaCaT cells, including cell swelling (area: 250.08 ± 17.28 μm^2^ vs. 135.78 ± 20.42 μm^2^, *p* < 0.0001) and irregular boundaries, indicate cytoskeletal disruption and potential apoptosis, consistent with *C. acnes* IA_1_–induced stress (Table 5). Fluorescence microscopy revealed biofilm formation by *C. acnes* IA_1_ on keratinocyte surfaces, supporting its role in persistent infection and chronic inflammation [10]. The significant increase in IL-6 secretion validates the transcriptomic upregulation of IL-6 signaling and aligns with hub gene functions, as HNRNPA2B1 and RBM39 regulate cytokine expression through splicing [22]. These findings highlight IL-6 as a pivotal mediator of acne inflammation, suggesting that targeting its upstream regulators or CLR signaling could mitigate *C. acnes* IA_1_–induced damage. Furthermore, the cutaneous microenvironment and its extracellular matrix (ECM) components play a crucial role in modulating inflammatory responses to microbial stimuli, such as *C. acnes* infection. Recent studies highlight that sulfated glycosaminoglycans (sGAGs), including artificially sulfated hyaluronan derivatives, can influence keratinocyte proliferation, differentiation, and inflammatory signaling within the ECM. These modified sGAGs incorporated into collagen-based artificial ECMs have been shown to promote keratinocyte growth and regulate immune cell responses under inflammatory conditions, potentially attenuating excessive inflammation while supporting tissue homeostasis. Such ECM modifications may offer insights into how the skin barrier and microenvironment mitigate pathogen-induced keratinocyte activation in acne pathogenesis, complementing the CLR-mediated pathways identified in this study [29,30,31].

Despite these advances, limitations must be acknowledged. The HaCaT cell model, while effective for studying keratinocyte responses, lacks the complexity of the in vivo skin microenvironment, which includes sebocytes, fibroblasts, and immune cells that modulate acne progression [28]. Focusing on a single *C. acnes* IA_1_ strain (CICC 10864) may not capture the diversity of phylotypes contributing to acne etiology, as other phylotype strains (e.g., IA_2_, IB) or clinical isolates exhibit distinct virulence profiles [21]. Additionally, the 24-h co-culture duration may not fully reflect chronic acne dynamics, where prolonged bacterial exposure drives sustained inflammation. Future studies should incorporate multi-omics approaches, such as proteomics and metabolomics, to validate hub gene functions and explore their interactions with other skin cell types. Clinical cohorts with diverse acne severities and *C. acnes* phylotypes could enhance translational relevance, while CRISPR–based gene editing of HNRNPA2B1 or RBM39 in skin models could clarify their causal roles in inflammation and comedogenesis [32].

Our findings pave the way for microbiome-targeted therapies, such as inhibitors of CLR signaling or RNA splicing modulators, to reduce reliance on broad-spectrum antibiotics, which exacerbate resistance [33]. For example, small-molecule inhibitors targeting HNRNPA2B1-mediated RNA processing could suppress inflammatory cytokine production, offering a precision medicine approach to acne [34]. These results also highlight the potential of hub genes as diagnostic biomarkers, enabling early detection of severe acne phenotypes through non-invasive skin biopsies [20]. By elucidating the molecular interplay between *C. acnes* IA_1_ and keratinocytes, this study advances our understanding of acne pathogenesis and supports the development of novel therapeutic strategies to improve clinical outcomes and reduce recurrence.

In light of these findings, the identified hub genes (HNRNPA2B1, HNRNPM, and RBM39) and the enriched C-type lectin receptor (CLR) signaling pathway offer promising avenues for novel therapeutic development. Targeting RNA splicing regulators such as these hub genes could inspire the design of small–molecule modulators or RNA–based therapies to attenuate inflammatory cytokine production and keratinocyte hyperproliferation in acne. Similarly, CLR pathway inhibitors may be formulated into topical agents to selectively dampen pathogen-induced inflammation without broad antimicrobial effects, reducing the risk of dysbiosis and antibiotic resistance. Beyond pharmacological targeting, biomaterial-based strategies—such as scaffolds incorporating microbiome-modulating probiotics or prebiotics—could restore skin homeostasis by promoting beneficial commensals while suppressing virulent *C. acnes* strains. Looking ahead, integrating transcriptomic biomarkers from this study with predictive modeling approaches (e.g., machine learning algorithms trained on multi–omics data) holds potential for personalized topical interventions, enabling early identification of high-risk patients and tailored treatment regimens to improve efficacy and minimize recurrence [28,35,36,37,38].

## 5. Conclusions

This study elucidates the molecular mechanisms of *Cutibacterium acnes* IA_1_ in acne pathogenesis through transcriptomic profiling and network pharmacology in HaCaT keratinocytes. We identified 769 differentially expressed genes, with hub genes HNRNPA2B1, HNRNPM, and RBM39 emerging as key regulators of RNA processing, sebaceous gland inflammation, and keratinocyte proliferation. These genes, validated by significant IL-6 upregulation (*p* < 0.01), are promising biomarkers for acne severity and potential therapeutic targets. The C-type lectin receptor (CLR) signaling pathway was significantly enriched, driving inflammatory responses via NF-κB activation, a critical factor in acne lesion chronicity. Experimental data confirmed morphological changes and biofilm formation, underscoring *C. acnes* IA_1_’s role in sustained inflammation. These findings highlight novel molecular targets for microbiome–specific interventions, reducing reliance on antibiotics prone to resistance. Future research should validate these biomarkers across diverse *C. acnes* phylotypes and in vivo models, incorporating multi-omics to enhance translational potential. Targeting HNRNPA2B1–mediated RNA processing or CLR signaling could lead to precision therapies, improving acne management and minimizing recurrence.

## Figures and Tables

**Table 1:** The top 10 upregulated and downregulated DEGs.

	Gene Name (Upregulated)	Gene Name (Downregulated)
1	*ITPRIP*	*PDE4D*
2	*SERPINF2*	*CCNC*
3	*TRAF5*	*CWF19L2*
4	*PIK3C2B*	*HNRNPA3*
5	*BICRAL*	*RRAS2*
6	*PSMD4*	*PPIP5K2*
7	*JADE1*	*PTGS2*
8	*CASP7*	*NDUFA5*
9	*TMPRSS4*	*ZDHHC6*
10	*RAN*	*RBM39*

**Table 2:** The top 10 GO terms for biological processes, cellular components, and molecular functions.

BP	CC	MF
interferon-mediated signaling pathway	nucleus	double–stranded DNA binding
negative regulation of DNA–templated transcription	nucleoplasm	DNA–binding transcription factor activity
negative regulation of RNA biosynthetic process	intracellular membrane–bounded organelle	transcription regulator activity
negative regulation of RNA metabolic process	membrane-bounded organelle	DNA-binding transcription factor activity, RNA polymerase II–specific
negative regulation of cellular metabolic process	actin-based cell projection	DNA binding
negative regulation of macromolecule metabolic process	nucleosome	transcription cis–regulatory region binding
regulation of multicellular organismal process	protein–DNA complex	transcription regulatory region nucleic acid binding
negative regulation of nucleobase-containing compound metabolic process	cytosol	sequence-specific double–stranded DNA binding
negative regulation of cellular biosynthetic process	histone acetyltransferase complex	sequence–specific DNA binding
negative regulation of metabolic process	acetyltransferase complex	RNA polymerase II transcription regulatory region sequence–specific DNA binding

**Table 3:** List of enriched pathways grouped into functional categories.

Functional Category	Representative Pathways	Adjusted *p*-Value	Number of Genes
Immune Response	p53 signaling pathway	<0.01	15
	Fanconi anemia pathway	<0.05	10
Chromatin Remodeling	Homologous recombination	<0.01	20
	Nucleotide excision repair	<0.05	18
Metabolic Processes	Ubiquinone biosynthesis	<0.05	8
	Aminoacyl-tRNA biosynthesis	<0.05	12
RNA Processing	Spliceosome	<0.001	25
	RNA transport	<0.01	22
Protein Degradation	Proteasome	<0.01	14
	Ubiquitin mediated proteolysis	<0.05	16

**Table 4:** Top 20 hub genes.

Top 20 Hub Genes	
*HNRNPA2B1*	*CPSF6*
*HNRNPM*	*HNRNPA3*
*RBM39*	*NONO*
*HNRNPU*	*SRRT*
*SRSF2*	*PABPC1*
*HNRNPH1*	*SF3B1*
*DDX39B*	*SF3B3*
*DDX5*	*NCL*
*SRSF6*	*BCLAF1*
*HNRNPR*	*LARP7*

**Table 5:** Comparison of area and circumference of HaCaT cells co-cultured with or without *C. acnes* IA_1_ (Mean ± SD, *n* = 10 technical replicates).

	Control of HaCaT Cells	HaCaT Cells Co-Cultured with *C. acnes*	*p*-Value
Area (μm^2^)	135.78 ± 20.42	250.08 ± 17.28	<0.0001
Circumference (μm)	46.72 ± 5.58	65.17 ± 3.42	<0.0001

**Table 6:** Number of bacterial cells associated with HaCaT cells (Mean ± SD, *n* = 3 three biological replicates).

Associated Bacterial Cells	Non-Associated Bacterial Cells
(4.03 ± 0.89) × 10^7^	(4.85 ± 0.59) × 10^9^

## Data Availability

The data presented in this study are available in the NCBI SRA database at https://www.ncbi.nlm.nih.gov/sra/PRJNA1199151 (accessed on 22 December 2025), accession number PRJNA1199151. Further inquiries can be directed to the corresponding author(s).

## Supplementary Materials

The following supporting information can be downloaded at: https://www.mdpi.com/article/10.3390/cimb48010034/s1.

## Abbreviations

The following abbreviations are used in this manuscript:

ITPRIP	Inositol 1,4,5-Trisphosphate Receptor Interacting Protein
SERPINF2	Serpin Family F Member 2
TRAF5	TNF Receptor Associated Factor 5
PIK3C2B	Phosphatidylinositol-4-Phosphate 3-Kinase Catalytic Subunit Type 2 Beta
BICRAL	BICRA Like Chromatin Remodeling Complex Associated Protein
PSMD4	Proteasome 26S Subunit Ubiquitin Receptor, Non-ATPase 4
JADE1	Jade Family PHD Finger 1
CASP7	Caspase 7
TMPRSS4	Transmembrane Serine Protease 4
RAN	RAN, Member RAS Oncogene Family
PDE4D	Phosphodiesterase 4D
CCNC	Cyclin C
CWF19L2	CWF19 Like Cell Cycle Control Factor 2
RRAS2	RAS Related 2
PPIP5K2	Diphosphoinositol Pentakisphosphate Kinase 2
PTGS2	Prostaglandin-Endoperoxide Synthase 2
NDUFA5	NADH: Ubiquinone Oxidoreductase Subunit A5
ZDHHC6	Zinc Finger DHHC-Type Palmitoyltransferase 6
RBM39	RNA Binding Motif Protein 39
RUNX1	RUNX Family Transcription Factor 1
TCF	Transcription Factor
TRAF3	TNF Receptor Associated Factor 3
IRF	Interferon Regulatory Factor
NFE2L2	NFE2 Like BZIP Transcription Factor 2
WNT	Wnt Family Member
HNRNPA2B1	Heterogeneous Nuclear Ribonucleoprotein A2/B1
HNRNPM	Heterogeneous Nuclear Ribonucleoprotein M
HNRNPU	Heterogeneous Nuclear Ribonucleoprotein U
SRSF2	Serine and Arginine Rich Splicing Factor 2
HNRNPH1	Heterogeneous Nuclear Ribonucleoprotein H1
DDX39B	DExD-Box Helicase 39B
DDX5	DEAD-Box Helicase 5
SRSF6	Serine and Arginine Rich Splicing Factor 6
HNRNPR	Heterogeneous Nuclear Ribonucleoprotein R
CPSF6	Cleavage and Polyadenylation Specific Factor 6
HNRNPA3	Heterogeneous Nuclear Ribonucleoprotein A3
NONO	Non-POU Domain Containing Octamer Binding
SRRT	Serrate, RNA Effector Molecule
PABPC1	Poly(A) Binding Protein Cytoplasmic 1
SF3B1	Splicing Factor 3b Subunit 1
SF3B3	Splicing Factor 3b Subunit 3
NCL	Nucleoli
BCLAF1	BCL2 Associated Transcription Factor 1
LARP7	La Ribonucleoprotein 7, Transcriptional Regulator
CASP1	Caspase 1
IKBKB	Inhibitor of Nuclear Factor Kappa B Kinase Subunit Beta
MAPK8	Mitogen-Activated Protein Kinase 8
