# Supplementary material for: Transcriptomic Profiling of Cutibacterium acnes IA1—Infected Keratinocytes Reveal Hub Genes and CLR Pathway in Acne Pathogenesis
Source: Curr Issues Mol Biol. 2025 Dec 26;48(1):34. doi: 10.3390/cimb48010034 (PMC12839951; doi:10.3390/cimb48010034)
Supplement: Supplementary file 1 [file cimb-48-00034-s001.zip › cimb-4062450-supplementary.pdf]

Table S1. Quality control metrics confirmed comparable sequencing quality between groups.

Sample	Group	Total Reads (million)	Mapped Reads(million)	Mapping Rate (%)
Control1	Control	46.65	45.62	97.80
Control2	Control	48.08	47.04	97.85
Control3	Control	41.92	41.21	98.31
C_acnes1	Infected	40.62	39.91	98.27
C_acnes2	Infected	44.94	44.06	98.04
C_acnes3	Infected	45.39	44.38	97.78
